# Supplementary material for: Identification of Freezing-Responsive microRNAs and Their Targets in Chinese Jujube by Small RNA and Degradome Sequencing
Source: Int J Mol Sci. 2026 Sep 4;27(17):7912. doi: 10.3390/ijms27177912 (PMC13566890; doi:10.3390/ijms27177912)
Supplement: Supplementary file 1 [file ijms-27-07912-s001.zip › Supplementary Material S2/novel_miR_36.pdf]

Provisional ID	:	novel_miR_36
Score total	:	37.1
Score for star read(s)	:	3.9
Score for read counts	:	35.3
Score for mfe	:	0.1
Score for randfold	:	-2.2
Score for cons. seed	:	
Total read count	:	81
Mature read count	:	80
Loop read count	:	0
Star read count	:	1

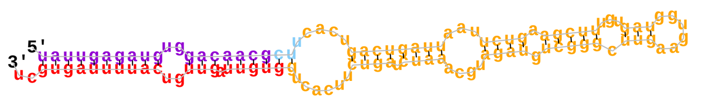

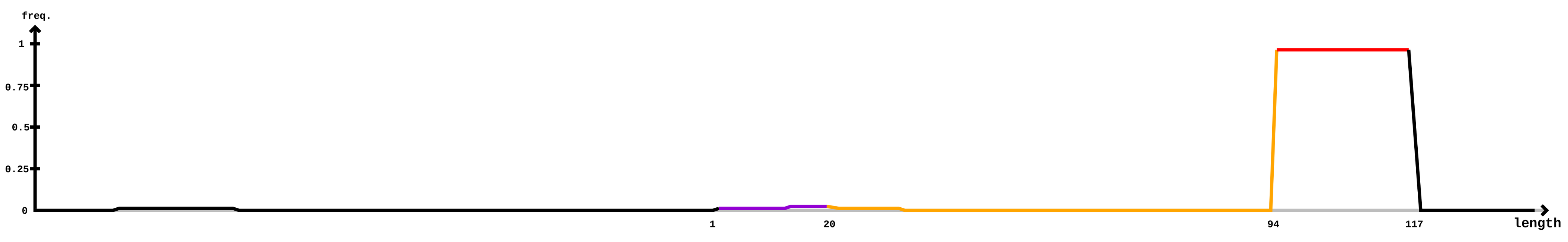

Star

Mature

```
5'- gguaccguucagauaucaauagagaaaagaggagagcaaagaagaaaccauacuggacacaauuuguaugcuguucaucugugaacuagaaucacaaguaucuccaugaaaauauugagauguggacaacgcuucacugacugauuaauucugaagcuuugugauggugaaguucgggcuguagaugcaaaucuagucuucacugguguuaguugucauuuuagugcuucuauaaacuauacguacauaa  -3'  obs
    gguaccguucagauaucaauagagaaaagaggagagcaaagaagaaaccauacuggacacaauuuguaugcuguucaucugugaacuagaaucacaaguaucuccaugaaaauauugagauguggacaacgcuucacugacugauuaauucugaagcuuugugauggugaaguucgggcuguagaugcaaaucuagucuucacugguguuaguugucauuuuagugcuucuauaaacuauacguacauaa       exp
    .((((.((..((......((((((......(((((...........(((...))).....((((((((.(((((((....))))).)).)).)))))).))))).......((((((((((..((((((((.....((((((((...((((.(((((...(((......))).))))).))))....)))).))))......)))))).)))..)))))))))).))))))...))..))))))....   reads  mm  sample
    ............auaucaGuagagaaaagagg..........................................................................................................................................................................................................................   1      1   LT-3
    ................................................................................................................uauugagaCguggacaacg.......................................................................................................................   1      1   LT-3
    ...........................................................................................................................gacaacgcuucacugacug............................................................................................................   1      0   JCK-2
    .............................................................................................................................................................................................................guguuaguugucauuuuagugcu......................   36     0   LCK-2
    .............................................................................................................................................................................................................guguuaAuugucauuuuagugcu......................   1      1   LCK-2
    .............................................................................................................................................................................................................gugGuaguugucauuuuagugcu......................   1      1   LCK-1
    .............................................................................................................................................................................................................guguuaguugucauuuuagugcu......................   40     0   LCK-1
    .............................................................................................................................................................................................................guguuaguugucauuuuagugcA......................   2      1   LCK-1
```
